# Morphology and Molecular Mechanisms of Hepatic Injury in Rats under Simulated Weightlessness and the Protective Effects of Resistance Training

**DOI:** 10.1371/journal.pone.0127047

**Published:** 2015-05-22

**Authors:** Fang Du, Ye Ding, Jun Zou, Zhili Li, Jijing Tian, Ruiping She, Desheng Wang, Huijuan Wang, Dongqiang Lv, Lingling Chang

**Affiliations:** 1 Department of Veterinary Pathology, Laboratory of Veterinary Pathology and Public Health,College of Veterinary Medicine, China Agricultural University, Beijing, China; 2 National Animal Protozoa Laboratory, College of Veterinary Medicine, China Agricultural University, Beijing, China; 3 State Key Laboratory of Space Medicine Fundamentals and Application, China Astronaut Research and Training Centre, Beijing, China; West Virginia University School of Medicine, UNITED STATES

## Abstract

This study investigated the effects of long-term simulated weightlessness on liver morphology, enzymes, glycogen, and apoptosis related proteins by using two-month rat-tail suspension model (TS), and liver injury improvement by rat-tail suspension with resistance training model (TS&RT). Microscopically the livers of TS rats showed massive granular degeneration, chronic inflammation, and portal fibrosis. Mitochondrial and endoplasmic reticulum swelling and loss of membrane integrity were observed by transmission electron microscopy (TEM). The similar, but milder, morphological changes were observed in the livers of TS&RT rats. Serum biochemistry analysis revealed that the levels of alanine aminotransferase (ALT) and aspartate aminotransferase (AST) were significantly higher (p<0.05) in TS rats than in controls. The levels of ALT and AST in TS&RT rats were slightly lower than in RT rats, but they were insignificantly higher than in controls. However, both TS and TS&RT rats had significantly lower levels (p<0.05) of serum glucose and hepatic glycogen than in controls. Immunohistochemistry demonstrated that the expressions of Bax, Bcl-2, and active caspase-3 were higher in TS rats than in TS&RT and control rats. Real-time polymerase chain reaction (real-time PCR) showed that TS rats had higher mRNA levels (P < 0.05) of glucose-regulated protein 78 (GRP78) and caspase-12 transcription than in control rats; whereas mRNA expressions of C/EBP homologous protein (CHOP) and c-Jun N-terminal kinase (JNK) were slightly higher in TS rats. TS&RT rats showed no significant differences of above 4 mRNAs compared with the control group. Our results demonstrated that long-term weightlessness caused hepatic injury, and may trigger hepatic apoptosis. Resistance training slightly improved hepatic damage.

## Introduction

Physical and psychosocial impacts on astronauts during space flight are always a major concern and also largely unknown, although humans have successfully explored space for over 50 years [1–3]. To understand the risks of spaceflight, ground-based simulated weightlessness models have played a significant role and have some advantages over spaceflight experiment [4]. Rat-tail suspension (TS) model has been used to study the effects of weightlessness on human body and organs by using simulated weightlessness condition for many years [5–7]. This model has demonstrated that long-term weightlessness can cause systemic organ damages, such as bone loss, bone fracture, muscle loss and atrophy, cardiovascular disorders, and renal dysfunction [8–13]. Simulated weightlessness can decrease hepatic glycogen and increase hepatic gluconeogenesis [14, 15]. Space flight can cause significant effects on liver glycogen, lipids, and enzymes in rats [16]. The levels of alanine aminotransferase (ALT) and aspartate aminotransferase (AST) increased after 7-day simulated weightlessness [17]. These data strongly suggested that weightlessness could cause hepatic injury.

Resistance training (RT) has been reported to be capable of protecting musculoskeletal system in various animal models. RT can improve bone strength by muscle contraction and stimulate bone formation [18]. Vibrations prevent the loss of strength in femur and tibia of adult rats [19]. However, it is unknown whether RT can protect hepatic injury caused by long-term weightlessness. Thus, this study was to evaluate hepatic injury associated with long-term weightlessness by TS model, hepatic injury improvement by TS&RT model, and possible molecular mechanism.

## Materials and Methods

### TS and TS&RT

Eight-week-old, male Wistar rats (approximately 290g) were bought from the Experimental Animal Center, Academy of Military Medical (certificate number: 038695). The rats were caged separately in a room maintained at 23C and controlled light/dark cycles (12 h/12 h). The rats were randomly assigned to three groups of 10 rats each as follows: control group (without tail suspension for 8 weeks), TS group, and TS&RT group. The treatments of rats in each group have been described previously [20]. Briefly, the hindlimb-unloading rats in the TS group were suspended using a tail cast device that contains an electrical stimulus apparatus, an indicator lamp, and a special orthostatic tube. The rats in TS&RT were forced to exercise by electrical pulse delivered by device that has one electrode attaching on rat’s tail and another electrode connecting to an aluminum plate under rat’s feet. The rats were trained to lift the inner cylinder using their shoulder to a preset height to turn the indicator lamp off. Otherwise, the device would deliver a 10 voltage of electrical pulse for 0.3 s to stimulate the rat to exercise. The rats developed a condition reflex to lift the cylinder ever other 2 s by following light-on and light-off signal. The rats performed four times (12 repetitions for each time) at 65% to 75% of 1 RM (the maximum weight lifted by a rat using the squat-training apparatus). Between every two times, the rats were allowed to rest in a standing position within the apparatus for 90 s. The RT was performed five days per week for 8 weeks. Prior to the application of tail suspension, the animals from the TS&RT group were acclimated to RT by using the above device to lift 50 g plus their own body weight.

### Specimen collections

After the two-month tail suspension, the rats were anesthetized with 1% sodium pentobarbital (45 mg/kg). Blood samples were collected from the abdominal aorta. Serum samples were separated to evaluate ALT, AST, and serum glucose levels. After animals were euthanized, livers were removed quickly and weighed. Fresh livers (about 20g) were collected and stored at -80°C for biochemical study. One piece of liver from each rat was collected and fixed in 2.5% (v/v) glutaraldehyde-polyoxymethylene solution for morphological and immunohistochemistry studies. All the experimental procedures were approved by the China Astronaut Research and Training Center Laboratory Animal Care Committee rules (ID: 26784) and the Institutional Animal Care and Committee of China Agricultural University (ID: 15883).

### Histopathological examinations

Completely fixed livers were embedded in paraffin blocks, sectioned at 4 μm, and stained with hematoxylin and eosin (H&E) by routine techniques. Additional unstained sections were used for immunohistochemistry, Mallory’s trichrome, Sirius Red, and Periodic Acid Schiff (PAS) staining. Hepatic morphology was evaluated with a light microscope.

### Transmission electron microscopy

The remaining tissues were post-fixed in 1% osmium tetroxide, embedded in Epon 812, sliced into 60-70-nm sections, stained with both uranyl acetate and lead citrate, and examined using transmission electron microscopy (TEM) (JEOL1230, JEOL, Tokyo, Japan) according to the method described by Ye Ding [21].

### Immunohistochemical assays

Primary antibodies used in this study were Bax (1:200 dilution; Boster CO., LTD, Beijing, China, BA0315), Bcl-2 (1:200 dilution; Boster CO., LTD, Beijing, China, BA0412) and active caspase-3 (1:200 dilution; Boster CO., LTD, Beijing, China, BA3968). Immunohistochemical staining was performed according to the Kit instructions (ZSGB-BIO, Beijing, China, SP-9001). Briefly, tissues sections were deparaffinized with xylene and rehydrated in ethanol series. Endogenous peroxidase activity was blocked by incubating sections in 0.3% H_2_O_2_ in methanol for 30 minutes. After applying blocking buffer (Zymed Laboratories, Inc. San Diego, USA), tissue sections were incubated with primary antibodies at 4°C overnight. Slides were incubated with a biotinylated anti-rabbit link secondary antibody (Beijing Zhong Shan Golden Bridge Biotechnology Co., Ltd. Beijing, China) for 30 minutes. Sections were then incubated with 3, 3-diaminobenzidine tetrahydrochloride (DAB) (Beijing Zhong Shan Golden Bridge Biotechnology Co., Ltd. Beijing, China) for 10 minutes and counterstained with Mayer’s hematoxylin.

Positive signals for Bax, Bcl-2 and active caspase-3 proteins were represented by a brown or yellow granular mass and were measured using the Motic Med 6.0 CMIAS Image Analysis System (Motic China Group Co., Ltd.). A total of 150 fields per rat (three fields per section, five sections per rat, 400× magnification for image analysis) were randomly selected and analyzed. The positive staining intensity was calculated as the ratio of the stained area to the total field assessed.

### Real-time PCR

TRIzol was obtained from Invitrogen, and the RNA transcription kits, DEPC, 2X Tap, PCR Mix, and DNA Marker were obtained from Applied Biosystems. The primer pairs used to analyze the cDNA are listed in Table 1 [22–24]. Real-time PCR was conducted as previously described [21]. For real-time PCR, 10 samples were used in each group, and each sample was run in triplicate.

**Table 1:** Primer sequences used for real-time PCR.

Sequence no.	Gene name	Primer sequence	Accession no.	Tm (°C)	Product size (bp)	Reference
1	Bax	F-ATGGAGCTGCAGAGGATGATT	NM017059	60	97	*Perez et al*[22]
		R-TGAAGTTGCCATCAGCAAACA				
2	Bcl-2	F-TGGGATGCCTTTGTGGAACT	U34964	60	73	*Perez et al*[22]
		R-TCTTCAGAGACTGCCAGGAGAAA				
3	Caspase-3	F-AATTCAAGGGACGGGTCATG	U49930	60	67	*Kijima et al*[23]
		R-GCTTGTGCGCGTACAGTTTC				
4	CHOP	F-CCAGCAGAGGTCACAAGCAC	NM007837	60	126	*Liu et al*[24]
		R-CGCACTGACCACTCTGTTTC				
5	GRP78	F-AACCCAGATGAGGCTGTAGCA	NM022310	60	91	*Liu et al*[24]
		R-ACATCAAGCAGAACCAGGTCAC				
6	JNK	F-TGATGACGCCTTACGTGGTA	XM341399	60	114	*Liu et al*[24]
		R-GGCAAACCATTTCTCCCATA				
7	Caspase-12	F-CACTGCTGATACAGATGAGG	NM130442	60	138	*Liu et al*[24]
		R-CCACTCTTGCCTACCTTCC				

### Quantitative analysis and statistical analysis

The experimental data were analyzed using a one-way ANOVA with the SAS statistical program, and multiple comparisons between the groups were performed using the S-N-K method (SAS Institute Inc., Cary, NC, USA). The results are expressed as the means and standard deviations (mean ± SD). P<0.05 was considered statistically significant. p<0.05 is indicated by “*” and “#”, whereas P<0.01 is indicated by “**” and “##”.

## Results

### Body and liver weights

As presented in Table 2, significant reduction of body weights in TS and TS&RT group were noted (p<0.01) and the liver weights in three groups showed no significant differences. Besides, the liver to body weight ratios of rats in TS and TS&RT group were significantly greater than in control group (p<0.05). However, there were no significant differences of liver to body weight ratios between TS group and TS&RT group.

**Table 2:** Body and liver weights.

	Control	TS	TS&RT
Liver weights (mg)	1244 ± 36.69	1258 ± 31.64	1152 ± 45.37
Body weights (g)	447 ± 24.59	379 ± 11.48**	366 ± 23.85**
Liver to body weight ratio (mg/g)	2.786 ± 0.10960	3.314 ± 0.1277*	3.143 ± 0.1330*

Data are shown as the means ± SD, n = 10.

* P <0.05

** P <0.01, significantly different from control group.

### Levels of ALT and AST in serum

**Fig 1:**
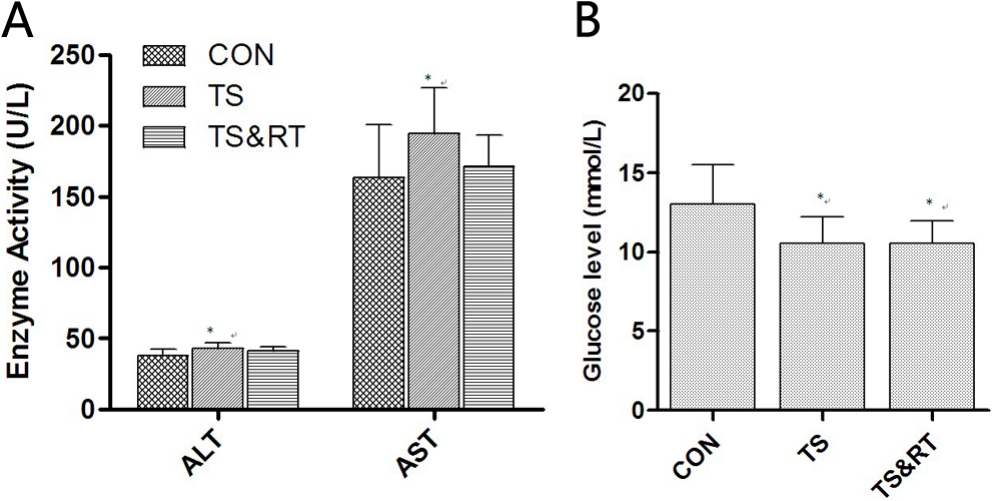
Levels of ALT, AST and glucose in serum. A: Serum ALT and AST levels: ALT and AST levels in the TS group were significantly increased compared with the control group, and ALT and AST levels in the TS&RT group were increased compared with the control group but reduced compared with the TS group; however, these differences were not significant. B: Serum glucose levels: serum glucose levels in the TS and TS&RT groups were significantly decreased compared with the control group (Data are expressed as the mean ± SD. * P <0.05, significantly different from control group).

The levels of ALT and AST in serum were presented in Fig 1A. Both enzymes in TS rats were significantly elevated compared with control group (p<0.05). However, the levels of ALT and AST in TS&RT group were between TS and TS&RT rats with no significance differences.

### Levels of serum glucose and liver glycogen

**Fig 2:**
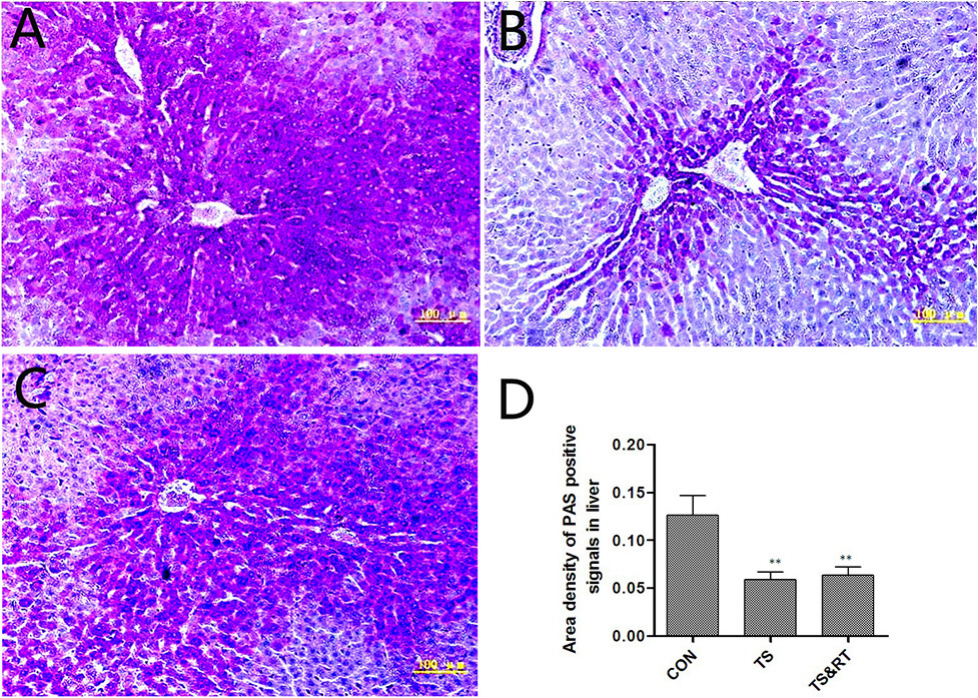
The analysis of liver glycogen by PAS staining. A-C: Liver glycogen as demonstrated by PAS staining (purple-red, 100×). A: Liver glycogen in the control group: large areas of hepatic cells around the central vein with positive PAS staining. B: Liver glycogen in the TS group: PAS-positive cells were significantly reduced, mainly at the edge of the portal area, with only scattered, sporadic purple liver cells surrounding the central vein. C: Liver glycogen in the TS&RT group: PAS-positive cells remained mainly in the area around the portal vein, and the number of PAS-positive cells increased around the central vein. D: A semi-quantitative analysis of liver glucose in the PAS-stained slides confirmed the results obtained for the serum glucose levels. Each value represents the percentage of the mean area of glycogen density in each group. n = 150 fields (10 rats) for each value. (Data are expressed as the mean ± SD. ** P <0.01, significantly different from control group.).

The levels of serum glucose in TS and TS&RT group were significantly decreased compared with control group (p<0.05) (Fig 1B). PAS staining demonstrated that the livers of control rats had strong PAS cytoplasmic stain (glycogen) in the hepatocytes around the central veins (Fig 2A). However, the livers of TS rats had a weaker PAS stain. Positive cells were mainly located at portal areas. Scattered PAS positive cells were noted at central vein areas (Fig 2B). In TS&RT group, PAS positive cells were located both in portal and central areas of livers (Fig 2C). A semi-quantitative analysis of liver glycogen by PAS staining (p<0.01) was consistent with the results of serum glucose (Fig 2D).

### Histological Examination

#### Light Microscopy

**Fig 3:**
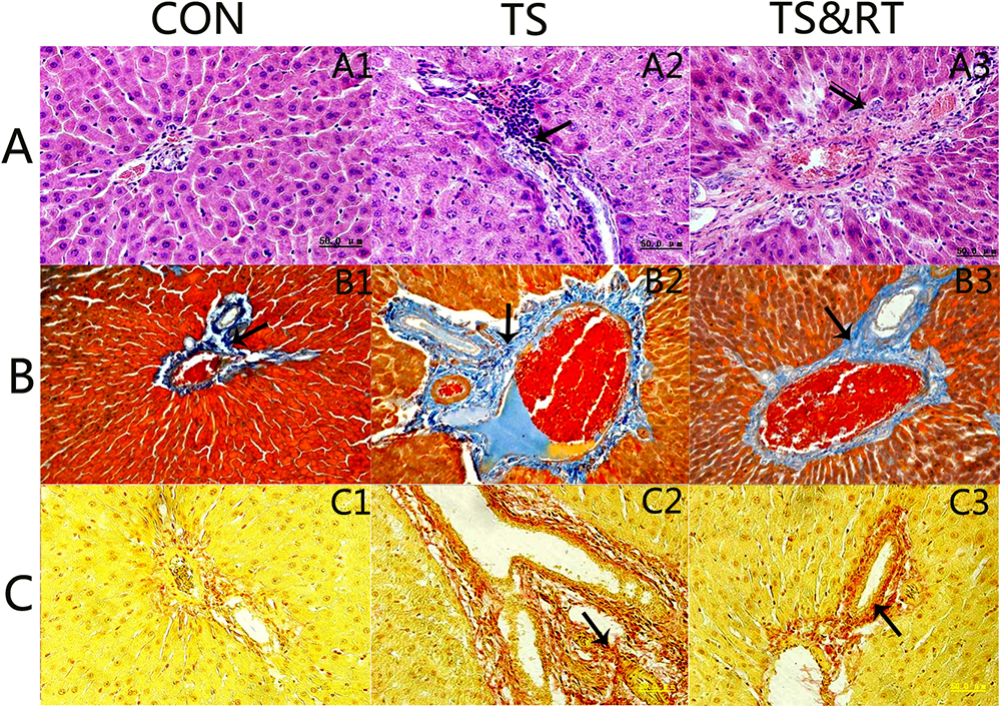
Histopathological analysis of rat liver paraffin sections stained with hematoxylin and eosin (H&E), Mallory’s trichrome stain, and Sirius Red stain. A: Morphological changes in the liver: the normal structure of the rat liver in the control group (A1). In the TS group, we observed mass granular degeneration and the swelling of hepatic cells around the central vein, vague cell boundaries, liver cell cord derangement, edema and the proliferation of bile ducts, and inflammation in liver portal areas (arrow) (A2). In the TS&RT group, pathological changes included the slight vacuolar degeneration of hepatic cells with a completed structure and clear boundaries and fibrosis in the portal areas of the liver in some of the rats (A3). B&C: Sections stained with Mallory’s trichrome (B) and Sirius Red (C) displayed various levels of the proliferation of fibrous liver tissue in the control (B1, C1), TS (B2, C2), and TS&RT groups (B3, C3). Collagen fibers stained blue with Mallory’s trichrome and red with Sirius Red stain (arrow). 400×

There were no morphological changes in the liver of control group (Fig 3A1). The livers of TS group had markedly hepatocyte degeneration characterized by swelling with large amount of cytoplasmic granules, especially at central areas (8/10), indistinctive cell boundaries (8/10), disorganized hepatic cords (6/10), bile duct proliferation (8/10), and chronic inflammation in portal areas (8/10) (Fig 3A2). The livers of TS&RT group showed slightly vacuolar degeneration, but normal structures of hepatocytes were preserved. Fibrosis in portal areas were noted in 2 animals from this group (2/10) (Fig 3A3).

Both Mallory’s trichrome and Sirius Red stain demonstrated that the livers of TS rats had significantly increased fibrosis in the lobular and central areas (Fig 3B2 and 3C2). However, this change was lower in the livers of TS&RT group (Fig 3B3 and 3C3). A semi-quantitative analysis of the staining intensity revealed an up to 6-fold increase in TS group compared with control group (p<0.01), and fibroplasia was 0.6-fold reduced in TS&RT compared with TS group (p<0.05) (Table 3). Mild fibrosis was also noted in the livers of control group (Fig 3B1 and 3C1).

**Table 3:** Semi-quantitative analysis of fibrosis by Mallory’s trichrome and Sirius Red staining.

	Mallory’s trichrome staining (%)	Sirius Red staining (%)
Control	2.15 ± 0.73	2.03 ± 0.43
TS	15.7 ± 3.38**	12.47 ± 2.11**
TS&RT	10.68 ± 2.01* #	7.82 ± 1.98* #

Data are expressed as the mean ± SD. Each value represents the percentage of the mean area of collagen density in each group. n = 150 fields (10 rats) for each value.

* P <0.05

** P <0.01, significantly different from control group.

# P <0.05, significantly different from TS group.

#### TEM observation

**Fig 4:**
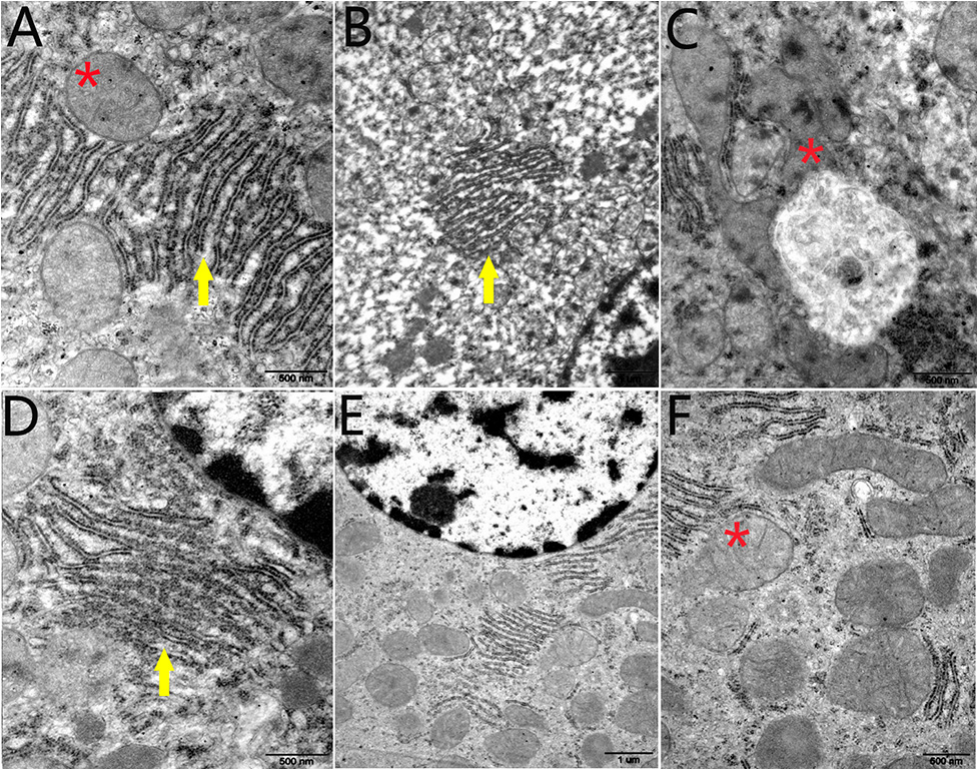
Transmission electron micrographs of liver in control, TS and TS&RT group. A: Liver tissue in control group, which was structurally intact with clearly visible mitochondrial cristae (star) and smooth endoplasmic reticulum cisternae (arrow). B-D: Liver tissue in TS group showed numerous mitochondria in hepatic cells with different degrees of swelling, and mitochondrial cristae displayed cavitation and even disappeared (star). The endoplasmic reticulum was expansive (arrow), and the liver cell matrix was loose. E, F: Liver tissue in TS&RT group showed that the degree of mitochondrial swelling and vacuolation was reversed (star), and the cytoplasmic matrix was more intact compared with TS group (A, C, D: 50000×; B, E: 20000×; F: 40000×).

The TEM examination of liver tissues revealed that liver tissues in control group were structurally intact with clearly visible mitochondrial cristae and smooth endoplasmic reticulum cisternae (Fig 4A). In comparison, different degrees of swelling in many mitochondria in hepatic cells, an occasional absence of cavitation in the mitochondrial cristae, an expansive endoplasmic reticulum (ER), and a loose liver cell matrix were observed in TS group (Fig 4B–4D). In TS&RT group, the degrees of mitochondrial swelling and vacuolation were reversed, and the cytoplasmic matrix was more intact than that in TS group (Fig 4E and 4F).

### Up-regulation of apoptosis-regulating proteins in TS rat livers

**Fig 5:**
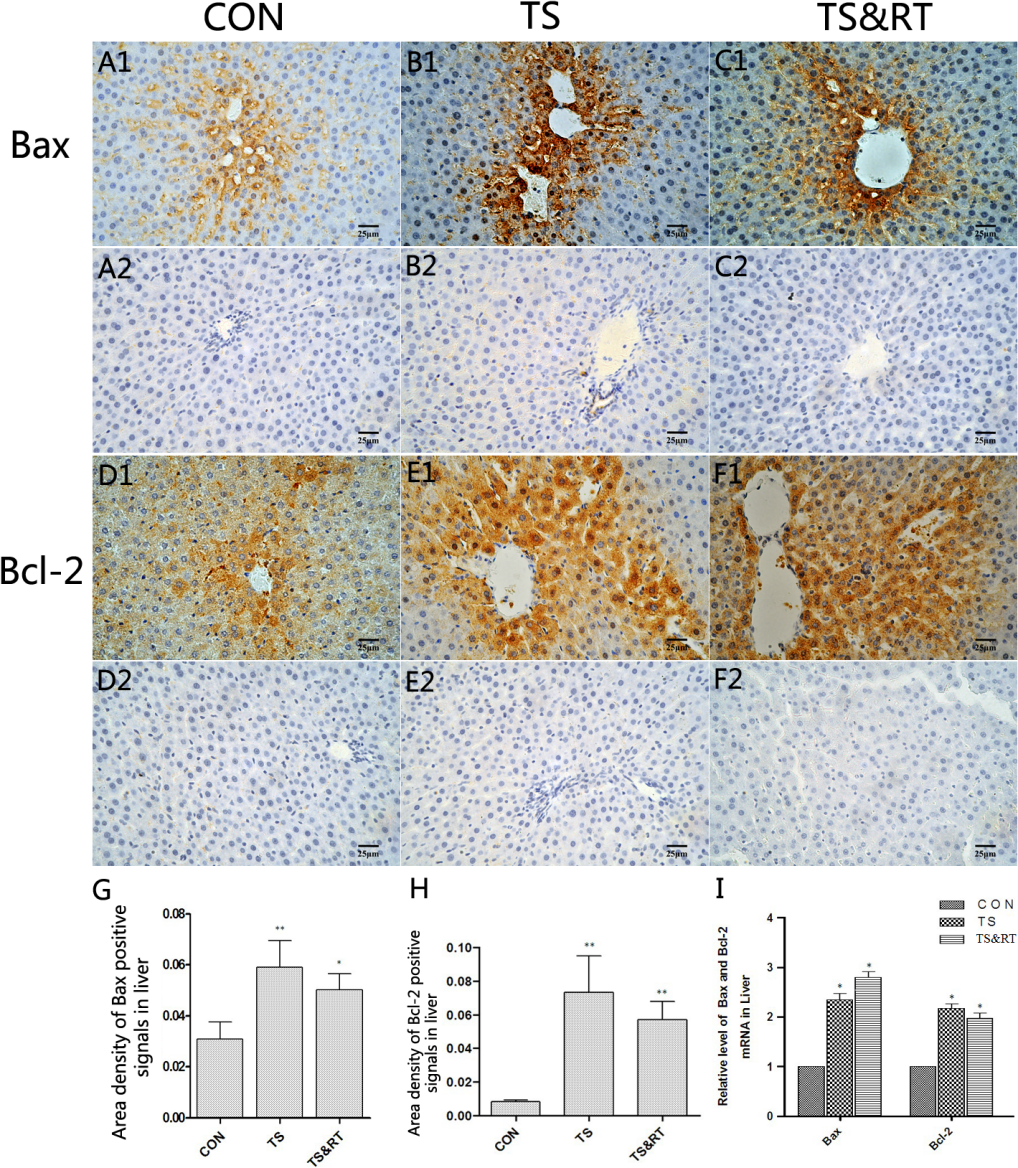
Immunohistochemical and real-time PCR analysis of Bax and Bcl-2. A1-C1: Histological sections of livers in control, TS and TS&RT group were stained with Bax antibody, and the positive signals were mainly located around the central veins in the livers. A2-C2: Histological sections of livers in control, TS and TS&RT group were stained with PBS to replace the Bax antibody as blank control; no positive signals were observed. D1-FI: Histological sections of livers in control, TS and TS&RT groups were stained with Bcl-2 antibody, and the location of the Bcl-2 positive signals was similar to the Bax positive signals. D2-F2: Histological sections of livers in control, TS and TS&RT groups were stained with PBS to replace the Bcl-2 antibody as a blank control; no positive signals were observed. G: A semi-quantitative analysis of the ratio of Bax positive staining to the total field. H: A semi-quantitative analysis of the ratio of Bcl-2 positive staining to the total field. I: Relative mRNA levels of Bax and Bcl-2. (Data are expressed as the mean ± SD. * P <0.05, ** P <0.01, significantly different from control group. 400×).

Immunohistochemical staining demonstrated the protein expressions of Bax and Bcl-2 in both TS and TS&RT rats were significant higher in central areas (Fig 5A1–5F1). The up-regulation of Bax and Bcl-2 protein expression was confirmed by Semi-quantitative analysis (* p<0.05, ** p<0.01) (Fig 5G and 5H). Real-time PCR showed that Bax and Bcl-2 mRNA expression of TS and TS&RT rats were significantly higher than that in control group (p<0.05), but Bax and Bcl-2 mRNA expression had no significance differences between TS and TS&RT rats (Fig 5I). Besides, Bax:Bcl-2 ratio was calculated approximately from the semi-quantitative analysis of these two proteins, as a result, 3 in control group, 0.85 in TS group, and 1 in TS&RT group, suggesting that Bax:Bcl-2 ratios in TS and TS&RT group were more lower than that in control group.

**Fig 6:**
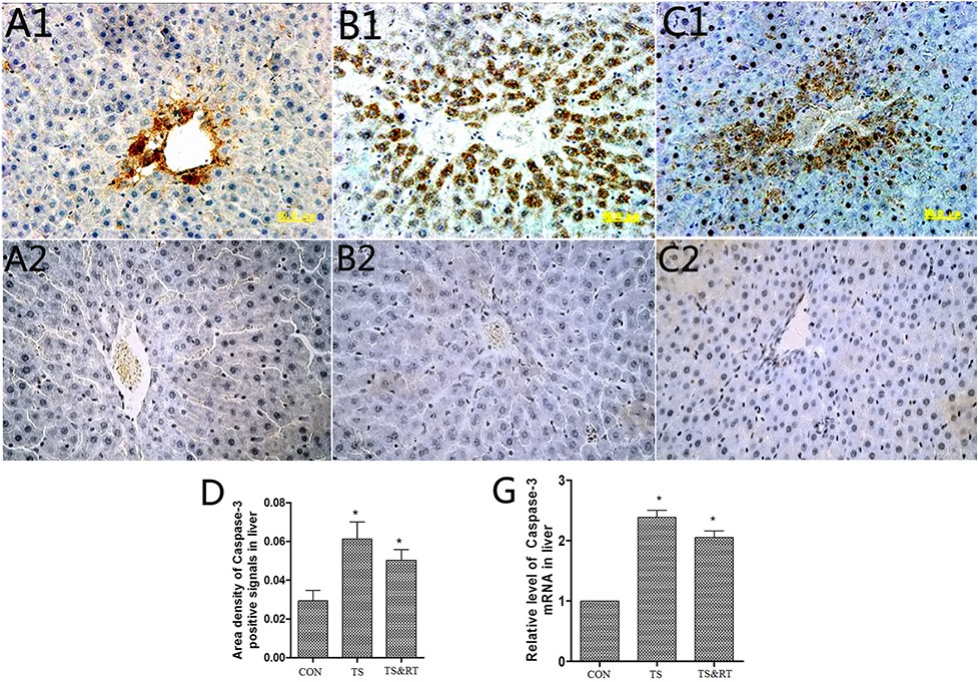
Immunohistochemical and real-time PCR analysis active of caspase-3. A1-C1: Histological sections of livers in control, TS and TS&RT group were stained with active caspase-3 antibody. A2-C2: Histological sections of livers in control, TS and TS&RT groups were stained with PBS to replace the caspase-3 antibody as blank control. D: A semi-quantitative analysis of the ratio of active caspase-3 positive staining to the total field. E: Relative mRNA levels of caspase-3 in control, TS and TS&RT rat livers (Data are expressed as the mean ± SD. * P <0.05, significantly different from control group. 400×).

The immunohistochemical study indicated that active caspase-3 was highly expressed in the liver of TS and TS&RT group (Fig 6B1 and 6C1), but it was lower in the livers of control group (Fig 6A1). These findings were consistent with a semi-quantitative analysis (p<0.05) (Fig 6D) and real-time PCR (p<0.05) (Fig 6E).

### Changes in ER-associated apoptosis proteins

**Fig 7:**
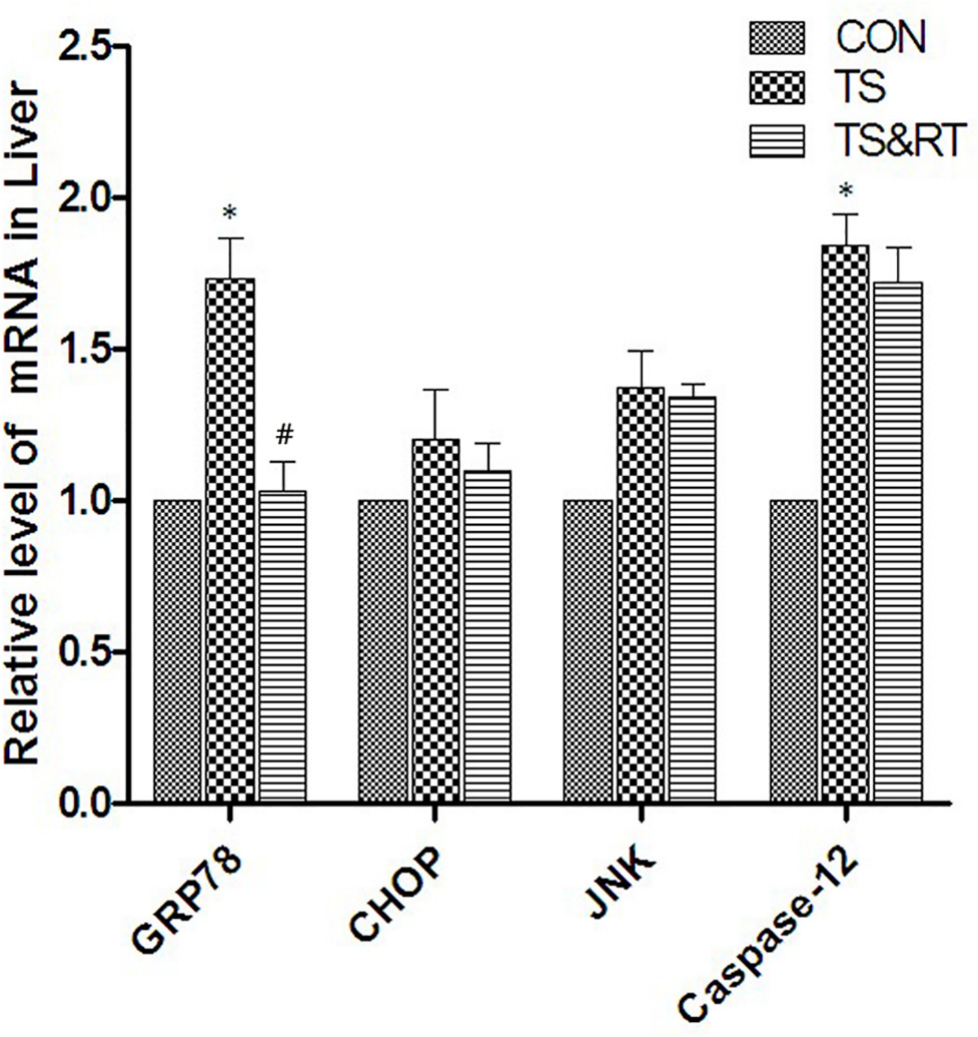
Real-time PCR analysis of changes in ER-associated apoptosis proteins. GRP78 transcription in TS group was significantly increased compared with control group, and GRP78 expression in TS&RT group was significantly reduced compared with TS group and similar to control group. Caspase-12 mRNA was significantly increased in TS group and decreased with no significant differences in TS&RT group. CHOP and JNK transcription was slightly increased in TS group compared with the control group, which was reduced compared with TS group, but no significant differences were noted (Data are expressed as the mean ± SD. * P <0.05, significantly different from control group. # P <0.05, significantly different from TS group.).

The real-time PCR results revealed that GRP78 transcription in TS liver was significantly increased compared with the control group (p<0.05). Furthermore, GRP78 mRNA in TS&RT group was significantly reduced compared with TS group (p<0.05). Caspase-12 mRNA was significantly increased (p<0.05), but CHOP and JNK transcription were slightly increased in TS group compared with control group. In TS&RT group, caspase-12, CHOP and JNK mRNA levels were lower compared with TS group, but no significant differences were noted (Fig 7).

## Discussion

Although studies showed that weightlessness could cause changes in hepatic metabolisms [14, 15, 25, 26], this study presented the evidences of hepatic alterations in morphology, enzymes, and molecular biology associated with long-term simulated weightlessness by rat tail-suspension model. Meanwhile, our results showed that the improvement of liver injury associated with weightlessness was minimal.

Long-term simulated weightlessness in this study increased liver to body weight ratio in both TS and TS&RT rats. This change was likely due to the loss of body weights rather than hepatic enlargement in TS and TS&RT rats. The rats in TS and TS&RT group had a significantly lower absolute body weights, but their absolute liver weights showed no significant differences. Body weight loss due to long-term simulated weightlessness had been documented previously [20], which is probably due to muscle atrophy, cardiovascular disorders, and other dysfunctions [8–13].

Long-term simulated weightlessness can cause changes in hepatic gluconeogenesis. The lower blood glucose and hepatic glycogen in TS and TS&RT rats in this study were consistent with previous studies [14, 26]. The alterations in glucose metabolism are associated with increased glycolysis, decreased fat oxidation, and accumulation of triglycerides and glycogen [26, 27]. Long-term simulated weightlessness can also cause hepatic damage. In this study, we not only confirmed that TS rats had significantly higher levels of AST and ALT in serum, but also, the first time, we presented morphological changes characterized by hepatocellular degeneration chronic hepatitis, and ER swelling and loss of membrane integrity transmission electron microscopically. Both space flight and simulated weightlessness can increase the levels of AST and ALT [17, 25]. Although the pathogenesis is unclear, our data suggested that high levels of AST and ALT in serum are due to hepatic injury caused by long-term simulated weightlessness. AST and ALT are liver enzyme markers, which indicate the health condition of hepatocytes [28]. When hepatocellular injury or death, release of hepatic enzymes from damaged hepatocytes increase levels of ALT and ALT in serum [29].

To understand the pathogenesis of hepatic damage caused by long-term simulated weightlessness, apoptosis associated molecules in liver had been measured in this study. Although there were no previous data of hepatic apoptosis associated with weightlessness, weightlessness had been reported to induce apoptosis in brain [30], muscle [31], and thyroid cells [32]. As Bax and Bcl-2 are pro-apoptotic and anti-apoptotic proteins, respectively, and the Bax to Bcl-2 protein ratio play an important role in the process of cell apoptosis, thereby shifting the balance between pro- and anti-apoptotic factors towards the anti-apoptotic side [33]. Under the experimental conditions considered in the present study, the expressions of Bax and Bcl-2 elevated, therefore the Bax to Bcl-2 ratios significantly decreased (0.85 vs 3 and 1 vs 3, respectively) in TS and TS&RT group compared with control group. However, this was not in accordance with the observation of *Nakamura et al*. [34] who reported that under gravity condition, in human osteoblastic cells, the expression of Bcl-2 was unchanged or even reduced, whereas the Bax expression increased in 24h, so the Bax to Bcl-2 ratio was higher. The differences may due to the short time in vitro of the previous study, and our research focused on the effects of long-time simulated weightlessness on rats in vivo, however, more researches were need to do to explain and confirm this.

Although it is unclear that the pathogenesis of cellular apoptosis caused by weightlessness. However, mitochondria damage demonstrated by electron microscopy (Fig 4C), the swollen mitochondria in the liver cells of TS rats indicating the occurrence of mitochondrial membrane damage, thus initiated the release of cytochrome c into the cytosol [33]. As a component of mitochondrial electron transfer chain, cytochrome c could initiate caspase activation after released from mitochondria, meanwhile, the recruitment of Bax into oligomers on the mitochondria then leads to permeabilization of mitochondrial outer membrane [35], which is considered one of the key control switches of apoptosis [31]. In the current study the elevation of active caspase-3, Bax, and Bcl-2 suggested that it may be initiated by the damage of mitochondria. Therefore, the results of this study might be related to the activation of the mitochondrial apoptosis pathway.

On the other hand, ER stress (ERS) induces apoptosis in multiple diseases [36–39]. TEM in this study showed ER degeneration in TS rats, which suggested ERS may also play an important role in the hepatocyte apoptosis. Several molecules, like CHOP, JNK, GRP78 and caspase-12, are involved in ERS-induced apoptosis [40, 41]. Our data demonstrated that the expression of GRP78 and caspase-12 were significantly increased in the livers of TS group. This result suggested that ERS-induced apoptosis may occur under simulated weightlessness.

Resistance training has been reported to improve bone strength [18, 19]. However, our study indicated that RT slightly compensated the liver injury caused by simulated weightlessness.

## Conclusions

Long-term weightlessness can cause liver damage characterized by hepatocytic degeneration, portal fibrosis, glycogen depletion, and mitochondrial and endoplasmic reticulum swelling and loss of membrane integrity. TS rats had significant higher levels of AST, ALT and glucose in serum than that in controls. High expressions and mRNA levels of Bax, Bcl-2 and active caspase-3 in TS rats suggested long-term weightlessness can induce hepatic apoptosis, likely through mitochondria pathway. RT exercises may reduce hepatic damage caused by long-term weightlessness, but its impacts were insignificant.
